# Differential recombination dynamics within the MHC of macaque species

**DOI:** 10.1007/s00251-014-0783-4

**Published:** 2014-06-17

**Authors:** Nanine de Groot, Gaby G. M. Doxiadis, Nel Otting, Annemiek J. M. de Vos-Rouweler, Ronald E. Bontrop

**Affiliations:** 1 Department of Comparative Genetics and Refinement, Biomedical Primate Research Centre, Lange Kleiweg 161, 2288 GJ Rijswijk, The Netherlands; 2 Department of Theoretical Biology and Bioinformatics, Utrecht University, 3584 CH Utrecht, The Netherlands

**Keywords:** MHC, Primates, Recombination, Microsatellites, Evolution

## Abstract

A panel of 15 carefully selected microsatellites (short tandem repeats, STRs) has allowed us to study segregation and haplotype stability in various macaque species. The STRs span the major histocompatibility complex (MHC) region and map in more detail from the centromeric part of the *Mhc-A* to the *DR* region. Two large panels of Indian rhesus and Indonesian/Indochinese cynomolgus macaques have been subjected to pedigree analysis, allowing the definition of 161 and 36 different haplotypes and the physical mapping of 10 and 5 recombination sites, respectively. Although most recombination sites within the studied section of the Indian rhesus monkey MHC are situated between the *Mhc-A* and *Mhc-B* regions, the resulting recombination rate for this genomic segment is low and similar to that in humans. In contrast, in Indonesian/Indochinese macaques, two recombination sites, which appear to be absent in rhesus macaques, map between the class III and II regions. As a result, the mean recombination frequency of the core MHC, *Mhc-A* to class II, is higher in Indonesian/Indochinese cynomolgus than in Indian rhesus macaques, but as such is comparable to that in humans. The present communication demonstrates that the dynamics of recombination ‘hot/cold spots’ in the MHC, as well as their frequencies, may differ substantially between highly related macaque species.

**Electronic supplementary material:**

The online version of this article (doi:10.1007/s00251-014-0783-4) contains supplementary material, which is available to authorized users.

## Introduction

The class I and II molecules encoded by the major histocompatibility complex (MHC) play a pivotal role in activating various adaptive immune-related reactions by presenting peptides to CD8^+^ or CD4^+^T cells, respectively. The MHC of primate species is one of the most gene-dense chromosomal regions, and the genes encoding peptide-presenting molecules are often characterized by a high degree of allelic heterogeneity (polymorphism) and copy number variation (diversity). Therefore, the MHC of two relevant model species, which are used to study human biology and disease—namely, the rhesus (*Macaca mulatta*) and the Indonesian/Indochinese cynomolgus macaque (*Macaca fascicularis*)—has been thoroughly investigated (Blancher et al. 2012b; Bontrop and Watkins 2005; Doxiadis et al. 2003, 2013; Karl et al. 2013; Li et al. 2012a, b; Liu et al. 2013; Mitchell et al. 2012; Otting et al. 2005, 2012; Zhang et al. 2012). Orthologs of the human (HLA) class II genes are also present in macaques, and most of them show a high degree of allelic variation (de Groot et al. 2012). However, some differences have been observed. For example, copy number variation in *DRB* genes is far more profound in macaques than in humans, whereas human populations seem to compensate for this by displaying extensive allelic heterogeneity. Equivalents of the *HLA-A* and *HLA-B* genes are present in macaques as well. The *Mhc-C* gene emerged in hominids (human/great apes) after the split from small apes and Old World monkeys and is, therefore, not present in macaques. However, this is compensated for by the fact that in macaques the *Mhc-A* and *Mhc-B* loci have been subjected to duplications and show copy number variation. On top of that, these *A* and *B* genes display transcription level polymorphisms as well. To simplify MHC-typing procedures, additional technologies to Sanger and next-generation sequencing (NGS) have been developed. Microsatellite loci or short tandem repeats (STRs)—repetitive sequence motifs of 2 to 6 bp—have been particularly useful to conduct disease association and population studies. Microsatellites have been used for molecular analysis of the HLA region, thanks to their characteristics such as a high degree of length polymorphism, chromosomal density, and Mendelian inheritance (Cullen et al. 2002; Foissac et al. 1997; Malkki et al. 2005; Martin et al. 1995a, b), and a database, *db*MHC, is available that records all progress (Gourraud et al. 2006). Microsatellites have been shown to be excellent tools for the quick and robust *DRB*, *A*, and *B* typing of macaques (Bonhomme et al. 2007; Doxiadis et al. 2007, 2009, 2011; Mee et al. 2009; Penedo et al. 2005; Wiseman et al. 2007; Wojcechowskyj et al. 2007). To refine further biomedical studies, additional microsatellite markers have been used to analyse the extended MHC region of macaques (Aarnink et al. 2013; Mitchell et al. 2012).

In humans, microsatellite markers have been used extensively to define recombination rates within the MHC, and it is proven that meiotic recombination does not occur randomly but seems to be restricted to specific chromosomal regions. Recombination frequencies defined by microsatellite analysis revealed that there are HLA regions—‘cold spots’—where no recombination is observed. In contrast, a lack of linkage between *TAP1* and *TAP2* alleles, for example, suggested the presence of a ‘hotspot’ (Martin et al. 1995a, b). More recent studies using sperm and SNP typing confirmed high linkage disequilibrium (haplotype blocks), which are often interrupted by recombination hotspots (Cullen et al. 2002; de Bakker et al. 2006; Walsh et al. 2003). In the present study, microsatellite analyses of the MHC of Indian rhesus and Indonesian/Indochinese cynomolgus macaques allowed us to map several recombination sites and suggest the existence of differential recombination rates and localizations of recombination ‘hot/warm’ and ‘cold/low’ spots in these two species.

## Materials and methods

### Materials

In the present study, 321 Indian rhesus macaques and 113 cynomolgus macaques of Indonesian/Indochinese origin were analysed. The animals have been housed at BPRC for more than five generations and are part of a self-sustaining breeding colony. Genomic DNA of the macaques was extracted from EDTA blood samples or from immortalized B cell lines, using a standard salting out procedure.

### STR genotyping

**Fig. 1:**
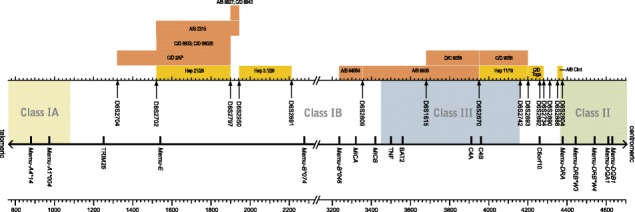
Mapping of recombination sites within the MHC of Indian rhesus and Indonesian/Indochinese cynomolgus macaques. The physical map is based on the work of Daza-Vamenta et al. (2004). The *Mamu-A*, *Mamu-B*, and *Mamu-DRB* alleles are named according to rules published in the IPD-MHC NHP database (de Groot et al. 2012). Recombination sites of rhesus macaques and cynomolgus macaques are color-coded (rhesus macaque: *brown*; cynomolgus macaque: *yellow*)

In the non-duplicated parts of the core MHC (*Mhc-A - DPB1*), human microsatellites were chosen, using the *db*MHC database (http://www.ncbi.nlm.nih.gov/gv/mhc/main.cgi?cmd=init). For >50 microsatellites, primers were developed according to the physical map of the rhesus MHC (Daza-Vamenta et al. 2004; Shiina et al. 2006), and the markers were tested for their suitability. Based on the degree of polymorphisms, heterozygosity, and their localization, 15 markers were chosen (Fig. 1, Table 1). The STRs were amplified by using a universal, fluorescently VIC-labeled primer adapter sequence (Applied Biosystems, Foster City, USA) together with the adapter sequence + unique 5′ primer and a unique 3′ primer (Invitrogen (Paisley, Scotland).

**Table 1:** Frequencies of microsatellites in Indian rhesus and Indonesian/Indochinese cynomolgus macaques

	Rhesus macaques (*N* = 321)			Cynomolgus macaques (*N* = 113)		
Locus	# of alleles	HObs	HExp	# of alleles	HObs	HExp
D6S2704	8	0.73	0.80	10	0.73	0.85
D6S2702	9	0.72	0.77	10	0.83	0.87
D6S2797	9	0.74	0.81	10	0.87	0.87
D6S2950	23	0.77	0.89	18	0.65	0.89
D6S2691	28	0.85	0.94	25	0.84	0.94
D6S2809	16	0.84	0.85	13	0.88	0.89
D6S1615	5	0.70	0.76	6	0.76	0.72
D6S2670	21	0.84	0.88	14	0.88	0.87
D6S2742	4	0.63	0.68	2	0.02	0.02
D6S2893	17	0.67	0.80	13	0.90	0.89
D6S2892	6	0.57	0.60	9	0.80	0.78
D6S2734	6	0.44	0.71	7	0.56	0.70
D6S2890	9	0.20	0.81	15	0.34	0.91
D6S2888	7	0.43	0.64	12	0.72	0.85
D6S2804	3	0.54	0.52	4	0.60	0.67
Mean #	11.4		0.76	11.2		0.78

Most primers could be used in a multiplex PCR reaction (Suppl. Table 1; primers 1 and 11, primers 5 and 13, primers 7 and 9, primers 8 and 15, and primers 12 and 14).

The PCR reaction was performed in a 25-μl reaction volume containing 1 unit of *Taq* polymerase (Invitrogen, Paisley, Scotland) with 0.1 μM of the unlabeled forward primer, 1.0 μM of the reverse primer, 1.0 μM of the VIC-labeled forward adapter primer, 2.5 mM MgCl_2_, 0.2 mM of each dNTP, 1× PCR buffer II (Invitrogen, Paisley, Scotland), and 10–25 ng DNA. For the multiplex PCR reactions, a second set of primers was added with the same concentrations.

The cycling parameters were a 5-min 94 °C initial denaturation step, followed by 5 cycles of 1 min at 94 °C, 45 s at 63 °C, and 45 s at 72 °C and then 25 cycles followed with 45 s at 94 °C, 30 s at 63 °C, and 45 s at 72 °C. A final extension step was performed at 72 °C for 30 min. The amplified DNA was prepared for genotyping according to the manufacturer’s guidelines and was analysed on the ABI 3130 genetic analyser (Applied Biosystems) with the GeneMapper software (Applied Biosystems).

### Allele frequency analysis

Allele frequency analyses of the macaque panels (Indian rhesus macaques, *N* = 321; Indonesian/Indochinese cynomolgus macaques, *N* = 113) were performed with the program Cervus 3.03 including 15 STR markers. For each locus, the number of alleles is shown together with the observed (HObs) and expected (HExp) heterozygosity and with the mean number of alleles and mean Hexp (Table 1).

### Simplification of *Mamu/Mafa-A*, *-B*, and *-DRB* haplotypes by “short” names

Complete *Mamu/Mafa-A*, *-B*, and *-DRB* haplotypes have been abbreviated to short names, mainly as described previously (Doxiadis et al. 2013; Karl et al. 2013). Briefly, the short names of the *Mamu/Mafa-A* and/or *Mamu/Mafa-B* haplotypes reflect one of the ‘major’ *A* or *B* transcripts (Karl et al. 2013). *Mamu/Mafa-DRB* haplotypes are named based on different region configurations ordered by increasing numbers of genes per haplotype (Doxiadis et al. 2013) (Suppl. Table 2)

### Founder haplotype definition and naming

**Fig. 2:**
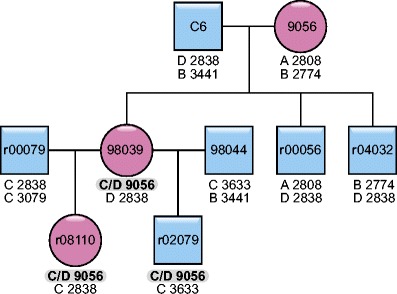
Example of a recombination event, which segregates in the family. The recombination event occurred in dame 9056 and is therefore called C/D 9056. It has been observed in the F1 and F2 generation in animals 98039 and r08110. Blue squares indicate males, red circles indicate females

**Fig. 3:**
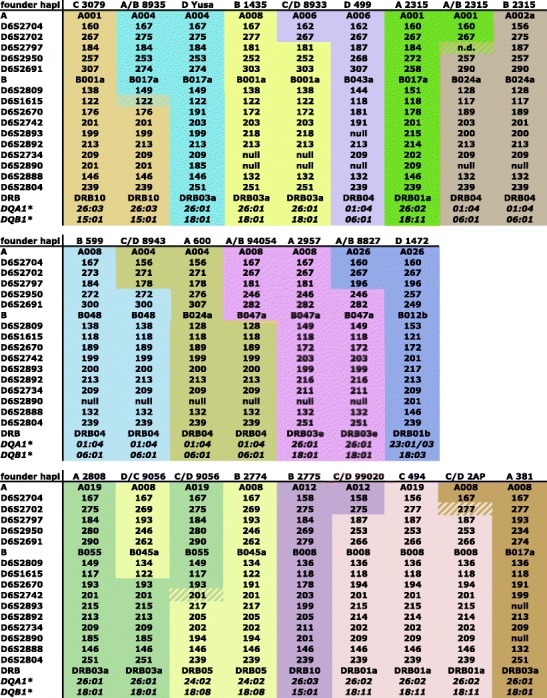
Recombinant and donor MHC haplotypes of Indian rhesus macaques. Donor haplotypes are color-coded, thus showing the recombination site in the recombinant haplotype. If a microsatellite is not informative for a certain recombination, this is indicated by *stripes*

**Fig. 4:**
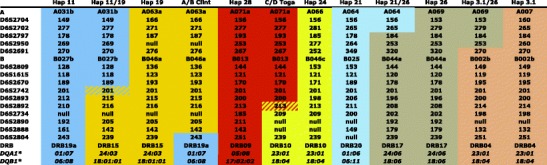
Recombinant and donor MHC haplotypes of Indonesian/Indochinese cynomolgus macaques. Donor haplotypes are color-coded, thus showing the recombination site in the recombinant haplotype. If a microsatellite is not informative for a certain recombination, this is indicated by stripes

Founder haplotypes have been defined as described earlier (Doxiadis et al. 2013). Haplotypes of Indian rhesus macaques were named according to the founder animal, with prefixes A and B indicating a founder male and prefixes C and D indicating a founder female. Haplotypes of Indonesian/Indochinese cynomolgus macaques, of which the founder animals could not always be defined unambiguously, were numbered as described earlier (Otting et al. 2012). Recombinant haplotypes were named according to the animal in which the cross-over event took place, with the prefixes A/B for a male and C/D for a female animal (e.g. C/D 2AP) (Figs. 1, 2, 3, and 4). If the recombination event could not be pinpointed to a certain animal, which is the case with some Indonesian/Indochinese cynomolgus monkeys because of unknown sires, the recombinant haplotype is named according to both donor haplotypes (e.g. Hap 11/19) (Figs. 1 and 4).

## Results

### Microsatellite analysis of the rhesus and cynomolgus macaque MHC region

Based on recorded microsatellite profiles in humans (Gourraud et al. 2006) (*dbMHC*/NCBI) and on the physical map of a heterozygous Indian rhesus macaque (Daza-Vamenta et al. 2004; Shiina et al. 2006), we have identified 15 different microsatellites covering the core MHC from the centromeric end of the class I *Mhc-A* to the *DRB* region of class II. Most of these microsatellites are mapping within the class III region and the adjacent section surrounding the C6orf10 locus (Fig. 1). Although the macaque *Mhc-A* and *Mhc-B* as well as the *Mhc-DRB* regions were subjected to contraction and expansion processes during their evolutionary past (Doxiadis et al. 2008; Kulski et al. 2004; Slierendregt et al. 1994), the genomic organization of the gene-rich regions in between has remained more or less intact (Daza-Vamenta et al. 2004; Watanabe et al. 2007; Yan et al. 2011). Therefore, the localization of the selected microsatellites in both macaque species is expected to be similar to that in humans.

In the first instance, microsatellite profiles of the Indian rhesus and Indonesian/Indochinese cynomolgus macaque panels were assayed to determine to what extent these STRs are polymorphic and can provide relevant information. The animals possess the founder MHC (*Mamu/Mafa-A*, *Mamu/Mafa-B*, *Mamu/Mafa-DRB*, *Mamu/Mafa-DQ*, *Mamu/Mafa-DP*) haplotypes, which have been described previously (Doxiadis et al. 2013; Otting et al. 2012). Most microsatellites display length polymorphism, and from 4 to 28 alleles with an expected heterozygosity (HExp) of >0.60 and a mean heterozygosity of >0.76 of all STRs, were detected (Table 1). Two markers do not fit in this schedule. Nevertheless, one of them, D6S2804, which displays a low level of polymorphism, has a HExp of >0.5 in Indian rhesus and 0.6 in Indonesian/Indochinese cynomolgus macaques and is therefore considered to be a useful marker. The second STR, D6S2742, displays polymorphism in our rhesus macaque panel but is dimorphic in Indonesian/Indochinese cynomolgus macaques, with one of the two alleles observed at a very low frequency (Table 1). The number of founder animals tested is far higher for the rhesus (*N* = 137) as compared to the Indonesian/Indochinese cynomolgus macaque colony (*N* ~ 30), and one may expect the number of STR alleles to be higher in the first species. Indeed, several microsatellite markers show more allelic variation in the Indian rhesus than in the Indonesian/Indochinese cynomolgus macaque panel. However, there are two microsatellites, which display far more polymorphism in Indonesian/Indochinese cynomolgus macaques (D6S2890 and D6S2888, Table 1), and the mean number of their alleles and expected heterozygosity levels is comparable. This finding is in agreement with earlier observations that Indian rhesus macaques show on average a lower degree of polymorphism for their MHC markers than other macaque species or rhesus macaques of other origins do (Doxiadis et al. 2013).

### MHC haplotype definition by microsatellite profiling

**Fig. 5:**
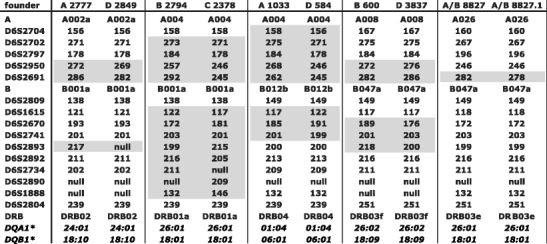
Examples of *Mamu-A/B/DRB/DQ*-identical haplotypes that can be differentiated by microsatellites. Microsatellites that show length variations are indicated by *grey boxes*

Based on STR profiles, 166 MHC founder haplotypes have been defined in the Indian rhesus macaque panel (see ‘Materials and methods’; Suppl. Table 3) (Doxiadis et al. 2013). Every STR founder haplotype profile was recorded in at least two related animals except those created by a recombination event. The profiles are listed together with their respective multi-locus MHC typing. Most founder STR profiles—namely, 161 out of 166—are unique. The few profiles, which are identical, most likely belong to related founder animals, originating from one breeding centre (Suppl. Table 3, black-bordered haplotypes). In addition, there are founder haplotypes that are identical for all their known MHC specificities, but can be distinguished based on STR patterns. Five examples are provided (Fig. 5).

Furthermore, *DRB* identical haplotypes are observed (1) to be mainly accompanied by the same *DQA1/DQB1* pair and (2) to segregate often with identical STR alleles, which map between the class III and II regions (Suppl. Table 3, colour-coded characters). The first observation confirms the tight linkage of *DR* and *DQ*, which is documented for several primate species (Begovich et al. 1992; Cullen et al. 2002; Doxiadis et al. 2000; Martin et al. 1995a, b). The second observation, however, may indicate low mutation rates of these C6orf10-surrounding markers in rhesus macaques and/or a tight linkage of the *DR* region not only with centromerically but also with telomerically located chromosome segments. This observation is notable for DRB04 haplotypes that are often accompanied by one specific D6S2742–D6S2804 pattern (Suppl. Table 3; D6S2742-199, D6S2893-200, D6S2892-213, D6S2734-209, D6S2890-null, D6S2888-132, D6S2804-239).

Additionally, length variation of one STR within a certain haplotype can also be observed in rhesus macaques. An example is provided by haplotype A/B 8827; as can be seen, the length of D6S2691 has changed from 282 to 278, as observed by segregation within its family (Fig. 5). Thus, a new STR allele has been ‘born’ and its birth could be recorded.

In our Indonesian/Indochinese cynomolgus macaque panel (*N* = 113), a total of 36 unique MHC STR profiles have been defined representing founder haplotypes. As for the Indian rhesus macaques, each STR profile has been detected in at least two related animals. All STR haplotypes have also been supplemented with relevant MHC typing (Suppl. Table 4), as published earlier (Otting et al. 2012). As in the rhesus macaque panel, we observed the appearance of a new STR allele (Suppl. Table 4, Hap 3 and 3.1), which differs in the length of marker D6S2691, and thus the potential birth of a new haplotype.

### Mapping recombination sites within the MHC of macaques

Within the Indian rhesus macaque haplotypes analysed (*N* = 648, Suppl. Table 3), we were able to define ten distinct recombination events, and the corresponding haplotypes segregate as stable entities in families (Figs. 2 and 3). According to the localization of the microsatellites, the recombination sites can be plotted on the physical map (Fig. 1). Most of the recombination events (i.e. six out of ten) took place between the *Mhc-A* and *Mhc-B* regions. More precisely, they map between the marker D6S2704, next to TRIM26, and D6S2950, which is situated in the middle between *Mamu-E* and the first *B* locus, Mamu-B*074:01. The other four crossing-over events are spread from the centromerical end of the *B* region to marker D6S2893, which is situated between class III and II, telomerically of C6orf10. Notably, two independent recombination events trace back to one female, 9056. One of the recombination events, C/D 9056, segregates for at least three generations, whereas the second one, D/C 9056, happened about 10 years later and has been observed in the first generation only. The second crossing-over event is nearly the complementary of the first one, and they can be mapped to both sides of the STR D6S2670, next to the *C4B* locus (Figs. 1 and 3).

A total of 225 haplotypes have been defined within our Indonesian/Indochinese cynomolgus macaque colony, and five crossing-over events have been observed (Fig. 4). Two recombinations could be designated to the animal in which the recombination took place, whereas this could not be determined for the other three recombinant haplotypes due to unknown sires. Since there are no STR length variations observed in the recombinant haplotypes in comparison to those of the ‘donor haplotypes’, it appears highly probable that all recombination events have happened within our colony, thus within the last five to six generations. Two recombination events are located between *Mhc-A* and *Mhc-B* regions as observed for most of the crossing-over events in the rhesus macaque. One of the crossing-over events, Hap 21/26, maps between markers D6S2702 and D6S2697 and the second one, Hap 3.1/26, between markers D6S2950 and D6S2691 (Figs. 1 and 4). Comparable to the rhesus macaques, one haplotype (haplotype 26) is involved twice in recombination events. However, in contrast to the observation in rhesus macaque 9056, the crossing-over events in the Indonesian/Indochinese cynomolgus haplotype 26 are not complementary and also—most probably—did not happen in the same animal. A third recombination, Hap 11/19, is identical to one in rhesus macaques, namely, C/D 9056. However, in Indonesian/Indochinese cynomolgus macaques, two recombinations, C/D Toga and A/B Clint, took place centromerically to marker D6S2893 within the region between class III and class II. More precisely, the recombination event in C/D Toga is localized between D6S2893 and D6S2734 surrounding C6orf10. The other crossing-over event can be pinpointed between two markers, D6S2804 and D6S2888, in A/B Clint (Figs. 1 and 4). To our knowledge, no recombination has been observed in this part of the chromosome in Indian rhesus macaques.

## Discussion

Profiling with 15 carefully selected STR markers in addition to typing for multiple MHC loci allowed us to define different haplotypes in great detail in large panels of Indian rhesus and Indonesian/Indochinese cynomolgus macaques. In such a way, even haplotypes that are identical for their ‘classical’ MHC alleles can be differentiated. Such a fine mapping may help to determine genes or genetic factors that are associated with susceptibility or resistance to diseases that are otherwise disguised by strong levels of linkage disequilibrium (LD) (reviewed in Trowsdale and Knight 2013).

In addition, the ‘birth’ of new haplotypes in both macaque colonies was recorded, as evidenced by the length change in one of the STRs (D6S2691). This marker represents a CCTT repeat of often more than 30 units and is therefore characterized by high levels of length variation. Although recent studies have shown that every microsatellite is unique in terms of its mutational variation, a relationship between motif length and mutagenesis has been observed (Eckert and Hile 2009). Thus, it is likely that a highly polymorphic microsatellite such as D6S2691 is prone to frequent mutagenesis. In the rhesus macaque colony, such an STR mutation was recorded; two generations after a recombination event had established a hybrid haplotype (Fig. 3, Suppl. Table 3: haplotypes A/B 8227 and A/B 8227.1). In the Indonesian/Indochinese cynomolgus macaque panel, the opposite event was observed. A new haplotype was first initiated by the birth of a new allele for marker D62691 (Suppl. Table 4: haplotypes 3 and 3.1). The subsequent recombination event happened one or two generations later (Fig. 4). Since the presence of specific sequence motifs such as GT_>12_ is significantly associated with recombination hotspots in the MHC, an STR-like D6S2691 may represent the substrate for recombination events (Cullen et al. 2002). Therefore, STR typing, especially for those with high repeat numbers, seems to represent an excellent manner for characterizing and studying the evolution of different DNA segments/haplotypes of highly variable regions such as sections of the classical MHC genes.

The analysis of 2,270 haplotypes in rhesus macaques (Doxiadis et al. 2013) and the data provided in this study show that Indian rhesus macaques have a comparable recombination rate for classes *A* and *B* (~0.26%), as observed in humans (~0.21 %) (Cullen et al. 2002; Martin et al. 1995a, b). Although the low number of informative meioses observed in a previous study (*N* = 332) (Otting et al. 2012) and in the present analysis may not allow statistically relevant conclusions, the recombination frequencies of the class I region in Indonesian/Indochinese cynomolgus macaques (~0.6 %) are apparently higher than those observed in humans and rhesus macaques. The difference between both macaque species becomes even more pronounced when recombination rates between the class III and II regions are taken into account. In this section of the MHC, no recombination has been documented in the rhesus macaque, and therefore, this part of the chromosome seems to represent a recombination desert. In contrast, in Indonesian/Indochinese cynomolgus macaques, two crossing-over events have been detected in this chromosomal section. Thus, in contrast to a recombination desert, which maps between C6orf10 and *DRA* in rhesus macaques, Indonesian/Indochinese cynomolgus monkeys apparently have one or more recombination warm/hot spots in this part of the genome. As a consequence, the mean recombination frequency of the core MHC, *Mhc-A* to class II (3.3 Mb), is also higher in our Indonesian/Indochinese cynomolgus macaque panel (1.5 % or 0.45 cM/Mb) than in Indian rhesus macaques (0.44 % or 0.13 cM/Mb), but is comparable to humans (0.49 cM/Mb) (Cullen et al. 2002).

Although at first glance the different recombination frequencies and cold/hotspots of the two related macaque species seem remarkable, comparable results have been reported in humans. Single-sperm typing of several humans has demonstrated that the distribution of recombination events may differ significantly between individuals (Cullen et al. 2002). Analyses of parent–offspring combinations of four human populations showed that linkage disequilibrium and recombination hotspots are population and haplotype specific (Ahmad et al. 2003; de Bakker et al. 2006). These results have been confirmed in a recent study comparing recombinations in the MHC of Asian, European, and African populations, which showed that >50 % of the recombination sites are unique for a single population (Lam et al. 2013). Taking into account that African and Europeans shared a common ancestor <250,000 years ago, whereas rhesus and cynomolgus macaque lineages separated ~1.3 million years ago, it seems plausible that substantial differences in recombination rates and sites are present in both species. It is noted that Mauritian cynomolgus macaques have a lower average recombination frequency (0.4–0.8 %) than the Indonesian/Indochinese cynomolgus macaques in our panel (1.5 %) (Blancher et al. 2012a). In this regard, the Mauritian cynomolgus monkeys resemble the Indian rhesus macaques. The low recombination frequencies in both populations are in accordance with a longer-ranged LD that seems to be more pronounced in bottle-necked populations, as has been documented for Mauritian cynomolgus and Indian rhesus macaques (Bonhomme et al. 2007; Doxiadis et al. 2003; Hernandez et al. 2007). However, MHC diversity after a bottle-neck may be partially restored by recombination events creating haplotypes that are composed of different MHC segments (Doxiadis et al. 2013; Karl et al. 2013; Wiseman et al. 2007). Similar observations have been made for West African chimpanzees, which experienced a selective sweep (de Groot et al. 2008). Certain forms of balancing selection may favour the selection of particular combinations of allotypes, thus actively selecting for recombinant haplotypes with novel combinations (de Bakker et al. 2006; Traherne et al. 2006). Since the Indonesian/Indochinese cynomolgus macaque colony studied was established with a relatively small number of founder animals (~30), it might be that such selection forces have been operative. At this stage, we do not understand whether this is due to pathogen-related pressure or whether this kind of selection takes place during stages of reproduction. The MHC in primates is considered to play a role in mate choice and reproduction (reviewed in Setchell and Huchard 2010; Ziegler et al. 2010). These ideas are supported by the observations that mate selection in most eukaryotic species is driven by some kind of self-/non-self-perception. Olfactory genes are localized in the extended MHC region, but many genes within the core MHC appear to play a role in reproduction, too (e.g. POU5F1, BAT3) (reviewed in Horton et al. 2004; Ziegler et al. 2010). Therefore, this panel of microsatellites may also help in understanding the role of the MHC region in reproductive biology and its success.

## Electronic supplementary material

Below is the link to the electronic supplementary material.

Suppl. Table S1 (DOC 45 kb)

Suppl. Table S2 (XLS 55 kb)

Suppl. Table S3 (PDF 1969 kb)

Suppl. Table S4 (PDF 1794 kb)
