# Supplementary material for: Differential recombination dynamics within the MHC of macaque species
Source: Immunogenetics. 2014 Jun 17;66(9):535–44. doi: 10.1007/s00251-014-0783-4 (PMC4156779; doi:10.1007/s00251-014-0783-4)
Supplement: Supplementary file 1 — (DOC 45 kb) [file 251_2014_783_MOESM1_ESM.doc]

Suppl Table 1. Primers used for STR genotyping

| **#** | **Primer name** | **Sequence 5’- 3’** | **Length** |
| --- | --- | --- | --- |
| 1 | 5’D6S2704_F  3’D6S2704_R | **CACCAGGAGCACGCAAGGAC**ACTCTGGAGGATGGGATATA  CTGGTCTACTGCTATAGGGT | **Mamu:154-171**  **Mafa:147-169** |
| 2 | 5’D6S2702_F  3’D6S2702_R | **CACCAGGAGCACGCAAGGAC**GCCTAAATGCTTCCTTGGATAT  AATAAAATCCAGGTCATGATGGAC | **Mamu:267-283**  **Mafa:263-283** |
| 3 | 5’D6S2797_F  3’D6S2797_R | **CACCAGGAGCACGCAAGGAC**GAGATCATGCCACTTCACTC  TGGGATCATACAACAGAGAC | **Mamu:172-196**  **Mafa:175-196** |
| 4 | 5’D6S2950_F  3’D6S2950_R | **CACCAGGAGCACGCAAGGAC**TGTGGGACTTTACAGCCTCC  GGCTGCAATGAGCTAGGATTG | **Mamu:234-334**  **Mafa:245-286** |
| 5 | 5’D6S2691_F  3’D6S2691_R | **CACCAGGAGCACGCAAGGAC**GTAGCTGTGGAAACAGTGTC  CCTTGACTTGAAACTCAGAG | **Mamu:241-325**  **Mafa:237-361** |
| 6 | 5’D6S2809_F  3’D6S2809_R | **CACCAGGAGCACGCAAGGAC**GAAGCCAGAAGTTCGAGACC  TAGCTGGGACTACAGACTGG | **Mamu:126-159**  **Mafa:128-155** |
| 7 | 5’D6S1615_F  3’D6S1615_R | **CACCAGGAGCACGCAAGGAC**CCTCTGCTCTCTGGGATTGC  GCCACTGCACTCAAGCCTT | **Mamu:117-122**  **Mafa:118-123** |
| 8 | 5’D6S2670_F  3’D6S2670_R | **CACCAGGAGCACGCAAGGAC**GTGACAGAGTGAGACCTTGTC  CTCATGTCCTCCACTAGAATC | **Mamu:155-197**  **Mafa:170-201** |
| 9 | 5’D6S2742_F  3’D6S2742_R | **CACCAGGAGCACGCAAGGAC**CAGTGACTTTGCTCTCAGCACC  AAGCCACTGCTCTCGTGCAG | **Mamu:191-203**  **Mafa:201** |
| 10 | 5’D6S2893_F  3’D6S2893_R | **CACCAGGAGCACGCAAGGAC**GTACACACCACCATGTCTGGCT  CAGGAGTACATGACCAGCAACA | **Mamu:198-222**  **Mafa:196-215** |
| 11 | 5’D6S2892_F  3’D6S2892_R | **CACCAGGAGCACGCAAGGAC**TGCATGTCCTGTGAGGTAAG  GCTGTTGTAGCACAAAAACA | **Mamu:205-227**  **Mafa:208-220** |
| 12 | 5’D6S2734_F  3’D6S2734_R | **CACCAGGAGCACGCAAGGAC**TTCAAGATTTCAGCTTGGTTC  GCTGGAGTTCTGCTAGGTTG | **Mamu:198-215**  **Mafa:185-209** |
| 13 | 5’D6S2890_F  3’D6S2890_R | **CACCAGGAGCACGCAAGGAC**GCCTAAGGTCTTCCAGGTTC  CTAGGTGTCCATCAGTGGATG | **Mamu:185-209**  **Mafa:185-211** |
| 14 | 5’D6S2888_F  3’D6S2888_R | **CACCAGGAGCACGCAAGGAC**TGTTGAGTTCTGGAGATAGG  TTGCCTATAGATGTTCCCTC | **Mamu:124-149**  **Mafa:132-179** |
| 15 | 5’D6S2804_F  3’D6S2804_R | **CACCAGGAGCACGCAAGGAC**GGTTCAATCTGCACATCTGG  TACCTCAGTCCTCCATCCCT | **Mamu:239-251**  **Mafa:231-251** |
|  | VIC-Adapter | **VIC-CACCAGGAGCACGCAAGGAC** |  |
