# Supplementary material for: Differential recombination dynamics within the MHC of macaque species
Source: Immunogenetics. 2014 Jun 17;66(9):535–44. doi: 10.1007/s00251-014-0783-4 (PMC4156779; doi:10.1007/s00251-014-0783-4)
Supplement: Supplementary file 3 — (PDF 1969 kb) [file 251_2014_783_MOESM3_ESM.pdf]

Supplementary Table 3. MHC founder haplotypes of Indian rhesus macaques

founder	Mamu-A	D6S2704	D6S2702	D6S2797	D6S2950	D6S2691	Mamu-B	D6S2809	D6S1615	D6S2670	D6S2742	D6S2893	D6S2892	D6S2734	D6S2890	D6S2888	D6S2804	DRB	DQA*	DQB*	# haplot
C 2378	A004	158	271	178	246	245	B001a	138	117	181	201	215	205	null	209	146	239	DRB01a	26:01	18:01	3
C 3005	A007	158	271	184	null	245	B001a	138	117	181	201	215	205	null	209	146	239	DRB01a	26:01	18:01	3
A Xusa	A008	167	277	184	261	278	B045a	134	122	189	201	215	205	null	209	146	239	DRB01a	26:01	18:01	2
B 2794	A004	158	273	184	257	292	B001a	138	122	172	203	199	216	211	null	132	239	DRB01a	26:01	18:01	2
C 2679	A003	158	267	184	257	324	B001a	153	120	197	201	215	214	202	209	146	239	DRB01a	26:02	18:11	4
D/C 3761	A008	167	269	181	257	284	B001a	149	117	176	201	215	214	202	209	146	239	DRB01a	26:02	18:11	3
A 2648	A007	156	267	184	257	307	B001a	140	120	186	201	215	214	202	209	146	239	DRB01a	26:02	18:11	2
C 494	A019	156	277	187	253	266	B008	136	118	194	201	215	214	202	209	146	239	DRB01a	26:02	18:11	3
C/D 2AP	A008	167	277	187	253	266	B008	136	118	194	201	215	214	202	209	146	239	DRB01a	26:02	18:11	2
C/D 99020	A012	158	275	187	253	266	B008	136	118	194	201	215	214	202	209	146	239	DRB01a	26:02	18:11	1
A 2794	A002a	158	275	184	257	292	B012b	149	122	172	201	215	214	202	209	146	239	DRB01a	26:02	18:11	4
C 3042	A011	158	267	193	257	290	B024a	128	120	178	201	215	214	202	209	146	239	DRB01a	26:02	18:11	2
D 3163	A002a	160	271	178	257	282	B024a	130	120	161	201	215	214	202	209	146	239	DRB01a	26:02	18:11	2
C 3034	A008	160	275	184	284	270	B055	138	117	178	201	215	214	202	209	146	239	DRB01a	26:02	18:11	2
C 1SA	A001	154	275	184	265	311	n.d.	149	117	176	201	215	214	202	209	146	239	DRB01a	26:02	18:11	2
A 2315	A001	160	267	184	272	258	B017a	151	118	178	201	215	214	202	209	146	239	DRB01a	26:02	18:11	2
D 597	A001	158	275	175	242	245	B012c	149	117	176	201	215	214	202	209	146	239	DRB01a	26:02	18:11	10
C 3620	A001	158	267	193	257	245	B012b	149	117	178	201	209	214	202	209	146	239	DRB01a	26:02	18:11	2
B EAW	A002a	156	271	178	246	245	B043a	144	118	193	203	199	214	202	209	146	239	DRB01a	26:02	18:11	10
C 426	A004	158	271	184	261	284	B048	138	122	193	203	199	214	202	209	146	239	DRB01a	26:02	18:11	7
D 2838	A224a	160	267	187	258	307	B045a	134	121	189	199	200	214	209	209	146	239	DRB01a	26:02	18:11	5
A 3026	A224a	160	267	187	258	307	B045a	134	121	191	199	200	214	209	209	146	239	DRB01a	26:02	18:11	4
D 2803	AUND	158	271	187	172	270	B043a	144	118	177	201	207	213	211	195	144	239	DRB03c	01:06	06:06	2*
D 1472	A026	160	267	196	257	249	B012b	153	121	172	201	217	213	209	201	146	239	DRB01b	23:01/03	18:03	5
A 2777	A002a	156	271	178	272	286	B001a	138	121	193	201	217	211	202	null	null	239	DRB02	24:01	18:10	10
D 2849	A002a	156	271	178	269	282	B001a	138	121	193	201	null	211	202	null	null	239	DRB02	24:01	18:10	3
B 2957	A002a	158	277	190	272	270	B001a	138	122	189	203	199	211	202	null	null	239	DRB02	24:01	18:10	9
C 3228	A002a	156	271	178	246	245	B012b/c	149	117	185	201	217	211	202	null	null	239	DRB02	24:01	18:10	2
A 2775	A019	156	277	187	272	274	B028	140	117	176	191	199	213	215	null	null	239	DRB02	24:01	18:10	2
C/D 8933	A006	162	267	181	252	303	B001a	138	122	172	203	218	213	null	null	132	251	DRB03a	26:01	18:01	1
B 1435	A008	167	277	181	252	303	B001a	138	122	172	203	218	213	null	null	132	251	DRB03a	26:01	18:01	5
C 3029	A002a	156	275	187	257	303	B001a	126	122	172	203	218	213	null	null	132	251	DRB03a	26:01	18:01	3
A 4043	A004	158	275	175	246	245	B012c	150	117	172	203	215	213	null	null	132	251	DRB03a	26:01	18:01	4
B 4032	A004	158	275	175	246	245	B012c	150	117	172	203	215	213	null	null	132	251	DRB03a	26:01	18:01	4
A 1435	A001	154	275	184	257	292	B048	138	122	172	203	215	213	null	null	132	251	DRB03a	26:01	18:01	14
A HQ	A004	154	275	184	257	292	B048	138	122	172	203	215	213	null	null	132	251	DRB03a	26:01	18:01	3
n.d.	A001	154	275	184	257	292	n.d.	138	121	172	203	215	213	null	null	132	251	DRB03a	26:01	18:01	2
C 3761	A012	162	267	181	246	282	B001a	138	122	172	203	215	213	null	null	132	251	DRB03a	26:01	18:01	2
D 2372	A001	158	275	187	253	278	B047a	126	121	172	203	215	213	null	null	132	251	DRB03a	26:01	18:01	3
D 2407	A008	154	269	184	257	282	B001a	151	121	161	203	215	213	null	null	132	251	DRB03a	26:01	18:01	8
D 2989	A004	158	267	172	null	262	B015a	149	122	191	203	215	213	null	null	132	251	DRB03a	26:01	18:01	2
D 3155	A007	156	267	175	334	278	B048	140	122	182	203	215	213	null	null	132	251	DRB03a	26:01	18:01	2
C 3633	A008	154	269	184	257	282	B001a	151	121	161	203	215	213	null	null	146	251	DRB03a	26:01	18:01	4
C 3837	A001	154	275	184	261	270	B001a	138	121	183	203	199	213	null	null	132	251	DRB03a	26:01	18:01	2
D Q068	A004	158	267	184	268	286	B001a	138	117	178	203	199	213	null	null	132	251	DRB03a	26:01	18:01	4
A 2808	A019	167	275	184	280	290	B055	149	117	193	201	215	213	209	185	146	251	DRB03a	26:01	18:01	2
B 2777	A019	167	275	184	284	290	B055	149	117	193	201	215	213	209	185	146	251	DRB03a	26:01	18:01	3
D/C 9056	A008	167	269	193	246	262	B045a	134	122	193	201	215	213	209	185	146	251	DRB03a	26:01	18:01	1
D 3509	A004	156	271	178	246	245	B012b	149	122	191	199	200	213	209	185	146	251	DRB03a	26:01	18:01	4
D Yusa	A004	167	275	184	253	274	B017a	149	122	191	203	199	213	209	185	146	251	DRB03a	26:01	18:01	17
C 3760	A011	154	275	184	250	270	B017a	153	122	172	203	215	213	null	185	132	251	DRB03a	26:01	18:01	2
C 2503	A008	154	275	184	257	286	B015a	149	117	178	203	199	213	198	207	132	251	DRB03a	26:01	18:01	3
C 3739	A008	167	275	187	253	270	B015a	159	117	189	203	199	213	198	null	132	251	DRB03a	26:01	18:01	4
A 381	A008	167	277	193	234	274	B017a	136	118	191	199	null	213	209	null	132	251	DRB03a	26:01	18:01	6
D 3617	A008	167	275	184	269	270	B048	140	122	161	203	200	216	211	null	132	251	DRB03a	26:01?	18:01	4
C 2829	A004	158	267	193	252	258	B045a	136	121	172	191	200	213	215	null	null	251	DRB03b	26:01	18:01	2
A 4032	A008	167	267	184	249	258	B017a	136	121	172	201	198	213	215	null	null	251	DRB03b	26:01	18:01	4
C 2836	A023	154	281	187	261	290	B066	153	121	193	203	199	213	209	185	146	251	DRB03b	26:01	18:01	2
D L61	A012	162	267	184	257	286	B043a	144	118	177	201	207	213	211	195	144	239	DRB03c	01:06	06:06	2*
D 2784	A002a	156	271	178	251	245	B047a	126	117	177	203	207	213	211	195	144	239	DRB03c	01:06	06:06	2
C 2803	AUND	154	275	190	242	278	n.d.	146	118	178	201	213	205	211	null	132	251	DRB01a	24:01	18:10	2
D 2559	A001	162	279	193	238	270	B017a	136	121	191	191	200	213	215	207	146	243	DRB03d	01:02	06:05	2
A 2957	A008	167	267	181	246	282	B047a	149	118	172	203	199	216	211	null	132	251	DRB03e	26:01	18:01	17
A/B 8827	A026	160	267	196	246	282	B047a	149	118	172	203	199	216	211	null	132	251	DRB03e	26:01	18:01	2
A/B 8827.1	A026	160	267	196	246	278	B047a	149	118	172	203	199	216	211	null	132	251	DRB03e	26:01	18:01	4
C 2414	A023	154	275	184	280	278	B012b	149	117	178	201	199	213	null	null	null	251	DRB03f	01:02	06:05	7
C 2419	A023	154	275	184	280	278	B012b/c	149	117	178	199	218	213	null	null	null	251	DRB03f	01:02	06:05	2
C Q068	A001	158	267	193	246	299	B001a	134	122	172	203	199	213	null	null	null	251	DRB03f	01:02	06:05	4
C 3044	A002a	156	271	178	272	282	B012b/c	149	120	193	203	199	216	211	null	null	251	DRB03f	01:02	06:05	2*
D 3600	A001	158	275	184	234	274	B017a	149	121	193	199	200	216	211	null	null	251	DRB03f	01:02	06:05	2
C 2560	A008	164	269	181	248	286	B047a	126	121	172	203	199	216	211	null	149	251	DRB03f	01:02	06:05	2
C 3162	A008	167	277	184	276	282	B024a	128	120	176	201	199	213	null	null	132	251	DRB03f	01:02	06:05	2
C 1644	A008	156	267	184	276	270	B006	134	122	176	null	199	216	211	null	132	251	DRB03f	01:02	06:05	2
D 2351	A004	156	271	178	246	245	B012c	149	122	192	null	222	213	211	190	146	243	DRB03f	01:09	06:09	3
D 2557	A008	167	275	193	261	307	B028	140	121	161	203	200	216	211	null	null	251	DRB03f	26:02	18:11	2
D 2505	A004	158	271	184	272	282	B047a	149	117	176	203	200	216	211	null	null	251	DRB03f	26:02	18:09	2
D 3837	A008	167	275	184	276	286	B047a	149	117	176	203	200	216	211	null	null	251	DRB03f	26:02	18:09	3
B 600	A008	167	275	184	272	282	B047a	149	117	189	201	218	216	211	null	null	251	DRB03f	26:02	18:09	3
C 3062	A007	154	275	184	272	286	B028	142	122	189	201	217	205	211	null	null	251	DRB03f	26:02	18:09	2*
A 3803	A008	167	275	184	265	282	B045a	134	122	189	199	199	216	211	null	132	251	DRB03f	26:02	18:11	2
C 2837	A001	158	275	175	242	278	B055	126	117	189	199	200	213	215	185	124	251	DRB03f	26:02	18:09	2
C 2838	A001	158	275	175	242	278	B055	126	117	189	199	200	213	215	185	124	251	DRB03f	26:02	18:09	3
D 594	A001	158	275	175	242	278	B055	126	117	189	199	200	213	215	185	124	251	DRB03f	26:02	18:09	2
D 669	A007	156	271	178	246	278	B055	126	117	189	199	200	213	215	185	124	251	DRB03f	26:02	18:09	2
C 3153	A004	158	269	193	248	258	B069a	138	122	172	199	200	213	209	null	132	239	DRB04	01:04	06:01	2
C 3403	A004	158	269	193	248	258	B069a	138	122	172	199	200	213	209	null	132	239	DRB04	01:04	06:01	2
A 3836	A004	167	269	193	248	258	B069a	138	122	172	199	200	213	209	null	132	239	DRB04	01:04	06:01	3
A 2774	A002a	158	275	193	265	245	B012b	149	122	191	199	200	213	209	null	132	239	DRB04	01:04	06:01	3
A H34	A001	158	275	181	272	303	B001a	138	118	189	199	200	213	209	null	132	239	DRB04	01:04	06:01	3
B 599	A008	167	273	184	272	300	B048	138	118	189	199	200	213	209	null	132	239	DRB04	01:04	06:01	2
C/D 8943	A004	156	271	178	272	300	B048	138	118	189	199	200	213	209	null	132	239	DRB04	01:04	06:01	1
C 1422	A002a	156	275	187	261	303	B015a	153	121	189	199	200	213	209	null	132	239	DRB04	01:04	06:01	2
A 600	A004	156	271	178	276	307	B024a	128	118	189	199	200	213	209	null	132	239	DRB04	01:04	06:01	26
A/B 94054	A008	167	267	181	246	282	B047a	128	118	189	199	200	213	209	null	132	239	DRB04	01:04	06:01	2
A EAW	A004	158	271	187	253	254	B015a	136	118	184	199	200	213	209	null	132	239	DRB04	01:04	06:01	22
B 3074	A012	162	279	190	261	245	B012b	140	118	189	199	200	213	209	null	132	239	DRB04	01:04	06:01	7
B 3441	A011	158	267	184	258	245	B008	136	122	193	199	200	213	209	null	132	239	DRB04	01:04	06:01	2
B 3643	A002a	156	269	187	257	290	B024a	126	117	193	199	200	213	209	null	132	239	DRB04	01:05:01	06:02	3
A 1033	A004	158	275	184	268	262	B012b	149	117	185	201	200	213	209	null	132	239	DRB04	01:04	06:01	2
A/B 2315	A001	160	267	n.d	257	290	B024a	128	117	189	203	200	213	209	null	132	239	DRB04	01:04	06:01	1
B 2315	A002a	156	275	187	257	290	B024a	128	117	189	201	200	213	209	null	132	239	DRB04	01:04	06:01	2
D 3061	A011	158	267	184	258	249	B008	136	122	193	203	200	213	209	null	132	239	DRB04	01:04	06:01	2
A 3441	A016	154	267	187	257	303	B024a	128	122	189	201	null	213	209	null	132	239	DRB04	01:04	06:01	11
B 3032	A002a	156	271	178	257	274	B001a	136	121	161	201	null	213	209	null	132	239	DRB04	01:04	06:01	2*
D 499	A006	162	267	187	268	307	B043a	144	118	181	191	null	213	209	null	132	239	DRB04	01:04	06:01	5
B 2521	A002a	156	271	178	276	282	B001a	138	121	185	203	200	213	null	207	146	239	DRB04	01:04	06:01	4
D 3136	A004	160	275	184	268	274	B001a	149	117	172	191	200	213	215	185	124	239	DRB04	01:04	06:01	2
D 432	A016	160	267	187	257	241	B008	136	117	178	203	200	213	209	192	132	239	DRB04	01:04	06:01	2
D 584	A004	156	271	178	246	245	B012b	149	122	191	199	200	213	209	null	132	239	DRB04	01:04	06:01	8
D 2822	A004	156	271	178	246	245	B012b	140	122	191	199	200	213	209	null	132	239	DRB04	01:04	06:01	2
D 4035	A008	171	275	184	257	299	B047a	149	117	176	201	217	213	209	null	132	239	DRB04	01:04	06:01	2
D 4033	A008	171	275	184	257	298	B047a	149	117	176	199	217	213	209	null	132	239	DRB04	01:04	06:01	3
C 3569	A004	158	275	187	null	278	B055	126	121	183	203	217	213	209	null	132	239	DRB04	01:04	06:02	2
C/D 9056	A019	167	275	184	280	290	B055	149	117	193	201	217	205	202	194	146	239	DRB05	24:02	18:08	4
B 2774	A008	167	269	193	246	262	B045a	134	122	191	201	217	205	202	194	146	239	DRB05	24:02	18:08	15
B H34	A023	154	275	181	246	257	B047a	149	117	172	201	219	205	211	194	146	239	DRB05	24:02	18:08	2
B 4043	A001	158	271	193	253	292	B012b	138	117	170	199	200	213	209	185	146	239	DRB05	24:02	18:08	3
D 2837	A004	156	271	178	246	245	B012b	153	117	176	191	199	213	215	203	149	251	DRB06	23:01	18:02	9
C 3477	A006	162	269	null	260	299	B024a	138	117	178	203	199	205	211	203	149	251	DRB06	23:01	18:02	6
D 3613	A006	156	281	193	257	274	B024a	128	117	189	201	222	205	211	203	149	251	DRB06	23:01	18:02	2
A 2522	A001	158	267	193	246	245	B012b/c	149	117	178	201	203	213	209	null	142	251	DRB06	23:01	18:02	2
B 4041	A008	167	275	184	280	300	B001a	138	122	172	201	null	213	null	207	146	251	DRB06	23:01	18:02	2
B 598	A002a	160	267	181	248	274	B017a	151	117	178	201	217	213	null	207	146	251	DRB06	23:01	18:02	2
D 3029	A008	160	267	181	248	282	B017a	151	117	178	201	217	213	209	207	146	251	DRB06	23:01	18:02	2
D 2801	A004	158	271	184	257	278	B001a	138	118	189	203	215	213	null	190	142	239	DRB07	26:03	17:05	4
A BXJ	A008	167	267	193	269	299	B069b	146	120	155	201	205	227	202	null	138	239	DRB08	24:05	15:03	21
A 3643	A006	156	269	181	246	270	B008	138	121	172	203	199	205	211	185	124	243	DRB09	26:02	18:11	4
C 2453	A004	156	271	178	246	245	B012b/c	149	122	189	203	199	205	211	185	124	243	DRB09	26:02	18:11	2
D 1425	A008	167	275	181	256	270	B028	134	122	191	203	199	205	211	185	124	243	DRB09	26:02	18:11	3
C 3033	A002a	156	275	187	257	298	B055	126	121	189	203	199	205	211	185	124	243	DRB09	26:02?	18:11	2
C 2857	A019	158	267	184	257	303	B028	140	118	187	199	199	205	211	185	124	243	DRB09	26:02	18:11	2
C 1425	A004	158	271	181	253	266	B001a	138	122	176	203	199	213	209	null	124	243	DRB09	26:02	18:11	4
C 3674	A001	158	275	187	246	274	B045a	134	121	191	201	215	213	209	201	146	239	DRB10	26:02	18:11	4
C 3079	A001	160	267	184	257	307	B001a	138	122	176	201	199	213	209	201	146	239	DRB10	26:03	15:01	9
A/B 8935	A004	167	275	184	253	274	B017a	149	122	176	201	199	213	209	201	146	239	DRB10	26:03	15:01	1
D 3079	A012	162	267	184	268	266	B001a	138	120	195	201	215	205	209	201	146	239	DRB10	26:03?	15:01	2
B 2775	A012	158	275	184	269	279	B008	136	118	178	203	199	205	211	201	146	239	DRB10	26:03	15:01	11
D 2817	A008	167	275	184	276	312	B043a	138	117	189	203	199	205	209	null	132	239	DRB10	26:03	15:01	2
C 2470	A008	167	275	184	265	300	B066	149	120	172	203	200	205	null	null	null	239	DRB11	05:04	17:08	3
A 3070	A007	160	267	187	257	null	B024a	128	122	189	201	218	205	null	null	132	239	DRB11	26:02	18:11	12
C 2753	A002a	158	277	175	null	290	B001a	138	117	189	201	217	213	null	207	146	243	DRB12	24:05	15:02	3
C 2354	A006	154	267	187	257	274	B069a	128	122	170	201	200	213	null	207	146	243	DRB13a	24:05	15:02	2
C 2006	A007	156	267	184	257	274	B047a	140	120	172	191	199	205	null	207	146	243	DRB13a	24:05	15:02	2
D 3114	A002a	156	271	178	246	303	B024a	128	121	189	null	220	205	null	207	146	243	DRB13a	24:05	15:02	2
D 3159	A011	160	267	193	257	278	B055a	126	117	178	201	215	213	215	207	146	243	DRB13a	24:05	15:02	2
C P48	A004	158	271	184	257	288	B069a	149	117	189	201	215	213	null	207	146	243	DRB13b	24:02	18:08	2
A 4041	A011	158	267	184	257	278	B017a	149	121	195	201	221	213	211	190	146	243	DRB14a	01:09	06:09	3
B 3070	A006	158	158	267	184	249	B008	136	122	189	201	221	213	211	190	146	243	DRB14a	01:09	06:09	4
B 3026	A001	156	275	193	257	296	B048	138	121	176	199	200	213	209	null	132	239	DRB15	01:05:01	06:02	8
A 2521	A006	158	275	184	265	282	B001a	138	117	161	199	200	213	209	null	132	239	DRB15	01:05:01	06:02	5
A HT18	A026	160	267	193	272	290	B001a	136	118	189	199	200	213	209	null	132	239	DRB15	01:05:01	06:02	2
C 2559	A012	162	271	181	260	307	B012b/c	149	122	189	199	200	213	209	null	132	239	DRB15	01:05:01	06:02	2
C 2613	AUND	158	271	172	246	274	B043a	144	118	161	199	200	213	209	null	132	239	DRB15	01:05:01	06:02	3
D 2987	A006	158	267	184	257	288	B015a	157	122	170	203	199	214	202	null	132	239	DRB15	01:05:01	06:02	4
B 1033	A002a	156	271	178	246	245	B012b/c	149	117	161	199	200	213	209	185	132	239	DRB15	01:05:01	06:02	2
D 2419	A008	158	275	184	248	286	B047a	149	117	178	201	206	213	209	203	132	239	DRB15	01:05:01	06:02	3
B 3836	A002a	167	275	184	253	325	B012b/c	149	117	178	201	220	213	null	null	132	239	DRB15	01:04	06:01	2
A 2807	A002a	158	277	175	null	290	B001a	138	122	172	201	198	213	207	185	124	239	DRB16	23:02	18:04	3
D 3739	A002a	158	277	175	null	290	B001a	138	122	172	201	198	213	207	185	124	239	DRB16	23:02	18:04	3
D 3238	A224a	167	275	193	269	274	B001a	138	122	172	201	198	213	207	185	124	239	DRB16	23:02	18:04	2
B 381	A025	167	269	193	253	254	B015a	157	122	172	201	198	213	207	185	124	239	DRB16	23:02	18:04	6
B HT18	A008	156	279	187	234	274	B017a	138	122	172	201	198	213	207	185	124	239	DRB16	23:02	18:04	2
C 2455	A008	167	283	184	265	300	B043a	144	118	189	201	215	213	207	185	124	239	DRB16	23:02	18:04	2
D 3760	A001	164	275	187	257	296	B048	134	122	161	201	215	213	207	185	124	239	DRB16	23:02	18:04	2
D 1478	A004	156	271	178	242	245	B012b/c	149	122	191	203	206	216	207	185	124	239	DRB16	23:02	18:04	2
D 3937	A008	156	275	187	257	290	B055	126	117	193	191	200	213	215	185	124	251	DRB17	26:02	18:11	3

648

*** implies that the haplotype is detected in two animals. The second haplotype of one animal, however, is observed only once or in a mixed bred animal and therefore not included in the dataset.**
