# Supplementary material for: Differential recombination dynamics within the MHC of macaque species
Source: Immunogenetics. 2014 Jun 17;66(9):535–44. doi: 10.1007/s00251-014-0783-4 (PMC4156779; doi:10.1007/s00251-014-0783-4)
Supplement: Supplementary file 4 — (PDF 1794 kb) [file 251_2014_783_MOESM4_ESM.pdf]

Supplementary Table 4. MHC founder haplotypes of cynomolgus macaques

Hapl	Mamu-A	D6S2704	D6S2702	D6S2797	D6S2950	D6S2691	Mamu-B	D6S2809	D6S1615	D6S2670	D6S2742	D6S2893	D6S2892	D6S2734	D6S2890	D6S2888	D6S2804	DRB	*DQA1**	*DQB1**	# hapl
1	A001	160	271	196	253	252	B044c	139	118	176	201	205	211	null	194	150	251	DRB21	*05:04*	*17:05*	**2**
2	A003	151	275	193	277	257	B044b	140	118	180	203	204	216	202	205	132	239	DRB13	*24:02:03*	*18:08*	**2**
3	A007	160	265	184	260	**266**	B002b	149	119	195	201	200	214	198	null	132	251	DRB04	*23:01*	*18:04*	**21**
3.1	A007	160	265	184	260	**270**	B002b	149	119	195	201	200	214	198	null	132	251	DRB04	*23:01*	*18:04*	**1**
3.1/26	A069	153	279	184	253	270	B002b	149	119	195	201	200	214	198	null	132	251	DRB04	*23:01*	*18:04*	**4**
5	A010a	160	279	190	261	329	B033	142	118	191	201	206	213	202	197	179	251	DRB03b	*01:14*	*06:23*	**4**
6	A010b	153	267	181	257	298	B103	136	119	176	201	201	213	202	188	146	243	DRB02	*05:07*	*17:06:02*	**6**
7	A018a	null	271	175	261	334	B002a	140	121	201	201	199	216	207	203	153	239	DRB14	*01:03:01*	*06:15*	**11**
8	A018	160	265	178	264	269	B090	149	118	170	201	211	210	null	191	161	243	DRB19a	*01:07*	*06:08*	**6**
9	A018b	156	275	184	256	314	B137	151	121	175	201	199	214	198	null	132	251	DRB04	*23:01*	*18:04*	**7**
10	A031a	149	271	181	249	293	B051	142	118	186	201	206	211	202	205	142	251	DRB11	*26:01:02*	*nd*	**6**
11	A031b	149	277	178	269	270	B027b	128	118	189	201	212	210	null	192	161	243	DRB19a	*01:07*	*06:08*	**3**
A/B Clint	A063a	166	271	187	null	276	B046a	136	123	193	201	215	216	null	205	142	243	DRB19a	*01:07*	*06:08*	**2**
11/19	A031b	149	277	178	269	270	B027b	128	118	189	201	215	216	null	205	142	239	DRB15	*24:03*	*18:01:01*	**1**
12	A031a	149	275	188	245	300	B027a	142	121	172	201	204	220	202	199	149	243	DRB16	*23:01*	*18:04*	**6**
13	A040	null	263	184	248	237	B069b	151	118	175	201	202	213	202	null	132	251	DRB06	*01:13*	*06:17*	**6**
14	A058	166	281	187	253	275	B007	149	121	197	201	196	213	202	202	161	243	DRB08	*05:02*	*17:02:01*	**11**
15	A059	149	273	181	261	290	B109	128	121	172	201	202	213	202	209	155	243	DRB22	*05:09*	*17:04*	**15**
16	A060	160	283	193	246	290	B036	157	119	172	201	206	213	null	205	149	251	DRB07	*05:03:02*	*16:01*	**2**
17	A061	158	273	193	249	null	B010	140	118	176	201	206	213	202	197	149	251	DRB19b	*01:06*	*06:11*	**8**
18	A062	160	275	190	249	278	B033	153	118	172	201	212	214	202	null	132	239	DRB09	*05:03:01*	*16:01*	**2**
19	A063a	166	271	187	null	276	B046a	136	123	193	201	215	216	null	205	142	239	DRB15	*24:03*	*18:01:01*	**8**
20	A063b	147	275	196	260	311	B011	142	121	172	201	200	214	202	null	132	239	DRB09	*05:03:01*	*16:01*	**5**
21	A064	156	265	178	264	349	B025	153	121	189	201	206	211	200	null	149	null	DRB20	*01:06*	*06:11*	**19**
21/26	A064	156	265	184	253	320	B044a	144	120	178	201	196	208	202	192	179	239	DRB17	*24:06*	*18:06*	**2**
23	A065	162	279	190	null	298	B021	155	122	172	201	211	216	202	205	142	239	DRB15	*24:03*	*18:01:01*	**5**
24	A066	156	281	185	277	252	B046c	153	121	171	201	198	213	209	null	null	239	DRB10	*23:01*	*18:04*	**17**
25	A068	151	263	190	253	361	B046b	136	122	172	201	199	214	204	null	132	239	DRB18	*01:02*	*06:07:01*	**8**
26	A069	153	279	184	253	320	B044a	144	120	178	201	196	208	202	192	179	239	DRB17	*24:06*	*18:06*	**3**
27	A070	158	279	181	272	289	B048	138	122	172	201	208	216	202	201	148	243	DRB05	*05:03:02*	*16:01*	**6**
28	A071a	156	277	193	253	267	B013	144	121	170	201	200	213	185	211	142	251	DRB09	*05:08*	*17:02:02*	**9**
C/D Toga	A071a	156	277	193	253	267	B013	144	121	170	201	200	213	209	null	null	239	DRB10	*23:01*	*18:04*	**1**
29	A071b	166	265	184	253	351	B104	136	119	176	201	196	208	null	209	134	239	DRB01	*24:02:01*	*18:08*	**2***
30	A072	169	273	184	249	267	B069a	146	119	172	201	202	217	202	null	132	239	DRB12	*05:03:01*	*16:01*	**7**
31	A092	147	271	181	286	null	B148	155	122	172	201	201	214	202	195	144	243	DRB03a	*01:08:02*	*06:01:02*	**5**
33^	n.d.	158	273	178	245	295	n.d.	153	120	172	201	196	219	202	185	146	231	DRB21	*05:04*	*17:05*	**2**
																					**225**

^ not in Otting et al., 2012; * implies that the haplotype is detected in two animals. The second haplotype of one animal, however, is observed only once and therefore not included in the dataset
